# Efficient Network Reconstruction from Dynamical Cascades Identifies Small-World Topology of Neuronal Avalanches

**DOI:** 10.1371/journal.pcbi.1000271

**Published:** 2009-01-30

**Authors:** Sinisa Pajevic, Dietmar Plenz

**Affiliations:** 1 Mathematical and Statistical Computing Laboratory, Division of Computational Bioscience, Center for Information Technology, National Institutes of Health, Bethesda, Maryland, United States of America; 2 Section on Critical Brain Dynamics, Laboratory of Systems Neuroscience, National Institute of Mental Health, National Institutes of Health, Bethesda, Maryland, United States of America; Indiana University, United States of America

## Abstract

Cascading activity is commonly found in complex systems with directed interactions such as metabolic networks, neuronal networks, or disease spreading in social networks. Substantial insight into a system's organization can be obtained by reconstructing the underlying functional network architecture from the observed activity cascades. Here we focus on Bayesian approaches and reduce their computational demands by introducing the Iterative Bayesian (IB) and Posterior Weighted Averaging (PWA) methods. We introduce a special case of PWA, cast in nonparametric form, which we call the normalized count (NC) algorithm. NC efficiently reconstructs random and small-world functional network topologies and architectures from subcritical, critical, and supercritical cascading dynamics and yields significant improvements over commonly used correlation methods. With experimental data, NC identified a functional and structural small-world topology and its corresponding traffic in cortical networks with neuronal avalanche dynamics.

## Author Summary

In many complex systems found across disciplines, such as biological cells and organisms, social networks, economic systems, and the Internet, individual elements interact with each other, thereby forming large networks whose structure is often not known. In these complex networks, local events can easily propagate, resulting in diverse spatio-temporal activity cascades, or avalanches. Examples of such cascading activity are the propagation of diseases in social networks, cascades of chemical reactions inside a cell, the propagation of neuronal activity in the brain, and e-mail forwarding on the Internet. Although the observation of a single cascade provides limited insight into the organization of a complex network, the observation of many cascades allows for the reconstruction of very robust features of network organization, providing valuable insight into network function as well as network failure. The current work develops new algorithms for an efficient reconstruction of relatively large networks in the context of cascading activity. When applied to the brain, these algorithms uncover the structural and functional features of gray matter networks that display activity cascades in the form of neuronal avalanches.

## Introduction

Cascade-like dynamics is characterized by the succession of events, or processes, that are causally related, and is frequently encountered in many complex systems (networks) across disciplines. For example, single cells in living organisms maintain metabolic, protein and gene-interaction networks with mostly unidirectional signaling cascades in which nodes represent metabolites, proteins and genes respectively [1]–[3]. At the next higher level of cell to cell interactions such as the brain, pyramidal neurons in the cortex connect with thousands of other neurons [4] thereby supporting cascades of neuronal activity in the form of waves [5], neuronal avalanches [6] and synfire chains [7],[8]. Cascade-like dynamics also occurs in many social networks such as the spread of epidemics [9] and gossip [10] in human networks as well as human travel itself [11]. This cascading dynamics carries the signature of the underlying statistical interdependencies between the interacting nodes, which are summarized by the functional network *topology*, represented by adjacency matrix indicating whether two nodes interact or not, and *architecture* [12], represented by a weighted graph which additionally indicates the magnitude of each interaction. The relationship between the cascading dynamics and the functional network is often poorly understood, even though reconstructing the network from the observed dynamics can provide crucial insights into the causal interactions between the nodes as well as the overall functioning of a complex system [13]. Of similar challenge remains the problem of how the functional architecture relates back to the structural organization of a network, that is to its physical nodes and physical connections between nodes [14]. While very similar dynamics can arise from fundamentally different network structures, e.g. for small neuronal networks with diverse elements [15], for large networks such as the human cortex the global brain dynamics has been shown to reflect fairly accurately the underlying structural connectivity, i.e. cortex anatomy [16],[17]. It is therefore critical to identify new approaches that provide insight into the functional and structural organization of a network based on the observed dynamics.

Correlations in the dynamics between nodes have been successfully used to identify functional links in relatively large networks such as obtained from MEG or fMRI recordings of brain activity (e.g. [18]–[20]). A pure correlation approach, however, is prone to induce false connectivities. For example, it will introduce a link between two un-connected nodes, if their activities are driven by common inputs [21],[22]. More elaborate approaches such as Granger Causality [23], partial Granger Causality [24], partial directed coherence (for a review see [25]), and transfer entropy [26] partially cope with the problem of common input, however, these methods require extensive data manipulations and data transformations and have been mainly employed for small networks [27],[28].

Here, we propose a new method that efficiently reconstructs the functional architecture of a network from the dynamics. In the theoretical part of the manuscript, we first introduce two different Bayesian approaches to reconstruct the network topology from the observed cascades: (1) the Iterative Bayesian (IB), and (2) the Posterior Weighted Averaging (PWA) with equal link priors. We then use PWA to derive the Normalized Count (NC) approach, a simple and efficient nonparametric algorithm that requires very little knowledge about the dynamical rules underlying activity cascades. We show that the NC, which is a hybrid between a Bayesian approach and a correlation method, performs almost as well as the IB when the exact probabilistic rules of the dynamics known. Using simulations, we demonstrate the utility of these algorithms for reconstructing random, small-world and scale-free network architectures from activity cascades modeled by subcritical, critical, and supercritical branching processes.

We apply our approach to neuronal avalanches, which are the activity cascades in the brain. It has been shown [6],[29],[30] that they spontaneously emerge in superficial layers of cortex, both *in vitro* (acute slices and slice cultures) [29]–[32] and *in vivo* [33]. They have also been demonstrated recently in the spike activity of dissociated cortex cultures [34],[35]. The network architecture that gives rise to neuronal avalanches is currently not known, although neuronal avalanches have been simulated in networks with scale-free [36],[37], fully connected [38], random [39], and nearest-neighbors [37],[40] topologies. Here we demonstrate a small-world functional topology of neuronal group formation in neuronal avalanches.

## Methods

### Theory

#### Bayesian network reconstruction from cascade dynamics

The cascade dynamics on a network can be described as a sequence of events 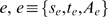, indicating the node, the time and the amplitude of an event respectively. We assume here that the observed sequence, 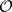, can be described by some underlying network structure, 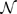, which we are trying to reconstruct, and certain probabilistic rules, 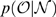. If 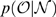 is known, then the most accurate reconstruction of 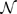 from the observed dynamics is obtained using the Bayesian approach [41]–[43], which relies on the Bayes rule
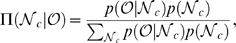
(1)where the index 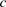 indicates a particular instance of network topology (adjacency matrix), 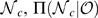 is the posterior probability of having 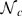, given the observation 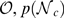 is the *a priori* (*prior*) probability, and 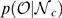 is the term that incorporates the above mentioned knowledge about the dynamics. The sum in the denominator is over all possible network configurations.

**Figure 1:**
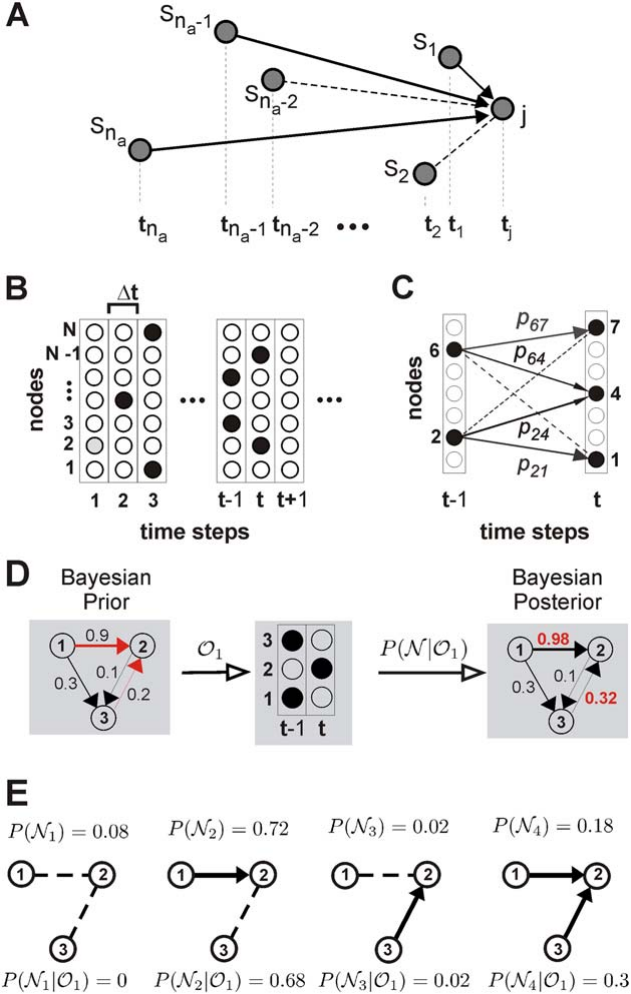
Bayesian network reconstruction from cascade dynamics. (A) The basic subnetwork motif for the Bayesian method consists of a target node 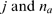 active source nodes 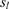 recorded as pairs 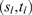 indicating the node and the time for each event. All possible network configurations 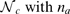 source nodes will be explored. A particular configuration with 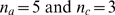 existing links is shown indicating that some of the active nodes will be able to directly influence the activation of the target node 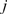 (solid line: directed link exists), whereas others will not (broken line: directed link absent). (B) Discretization of the cascade dynamics in time steps of length 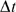 on a network with 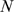 nodes (active nodes labeled black). (C) A branching process on a network is simulated by assigning activation probabilities to each outgoing link, here between source nodes 2 and 6 and target nodes 1, 4, and 7 (broken line: link absent). (D) An example of a Bayesian estimation given the observation 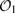 on a 3 node network (STES). Numbers indicate link activation probabilities. Red: links that will be modified given the target node 2. (E) The four configurations, 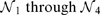, when the node 2 is the target node, showing their prior and posterior probabilities for the observation 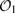 shown in (D). The posterior probabilities are used to calculate new link priors in the next estimation step by summing all posterior configuration probabilities, 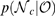 for those configurations containing the specific link 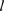. For example, only the second and fourth configuration posterior probabilities are added for the link 1→2.

Exploring all possible topological configurations for a complete network with 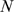 nodes is a daunting task, since that number is on the order of 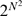, making this approach computationally intractable. To reduce the problem, we assume that the activation of a given target node 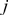 (*descendant*; see Figure 1A) can be caused only by a finite set of events 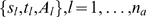, occurring on source nodes 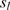 (*ancestors*) at prior times. The index 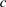 now enumerates all link configurations (topologies) by which the 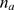 active source nodes can connect to the target node 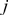. This reduces the number of configurations to be explored to 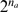, where 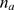 is the number of active source nodes considered. Thus, when exploring a particular topological configuration 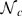, the activation depends only on 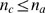 active nodes that connect to the target (see example in Figure 1A with 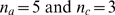). The number 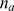 is not fixed and changes in time as different target nodes are explored. The 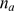 relevant ancestors are usually obtained using a cut-off time difference beyond which the activation of the target is impossible or unlikely. When the cascade dynamics is recorded in the form of a raster (see Figure 1B), the event times 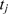 are discretized and events are placed into bins of fixed duration 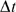, which allows a fixed number of preceding bins to be used in order to determine the 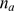 relevant source nodes. A single observation 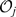 then reduces to a statement that an event 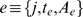 occurred on the target node 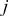, given the set of ancestor events 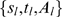, or, for binned data, 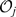 can also state that a node 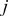 was not active within a given time bin 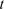. Thus, the reconstruction of the whole network, 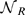 is subdivided into many simple Bayesian estimation steps focusing on a single target node and its corresponding subset of source nodes. We call this a single target estimation step (STES). To obtain 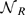 we combine these simple STES using two approaches that differ mainly in the way of handling the priors: (1) the *Iterative Bayesian* (IB), which starts with equal priors and builds them iteratively, and (2) the *Posterior Weighted Averaging* (PWA), for which the case of equal prior probabilities for link existence is explored. Both approaches are described in detail following the description of the dynamical model that we use in this work as an example, thus obtaining the dynamics term 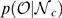 in Equation 1. Finally, we derive a nonparametric method based on PWA, that we call *normalized count* (NC) approach, to be used for reconstructing networks from point process dynamics.

#### Cascade dynamics: branching point process on a network

For many dynamical processes on a network it is reasonable to assume that the activation of a target node, 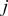, depends only on a finite set of prior events 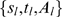. Then, 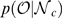 can be written as a general function of the event times, 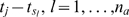, amplitudes 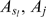, as well as 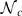 and other parameters needed to describe the dynamics.

In this work we focus on a specific type of cascade dynamics, i.e., a branching point process in which the probability 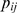 that a given network node 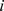 will activate node 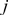 is fixed (Figure 1C). The branching process is specified in the form of a directed weighted graph with weights equal to 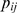 thus forming the network architecture [12]. The network topology is defined by the links which have non-zero probabilities of activation 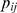. Depending on the values of 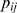, subcritical, critical and supercritical regimes can be observed for many network topologies.

Given heterogeneous probabilities 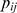 for each source node 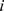 to activate the target node 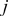, and the net configuration 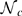 in which 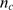 links from the 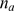 active nodes exist 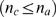, the probability that the node 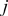 is active at time 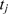 is
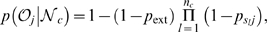
(2)where 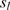 indicates the node index for the 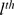 active source node, and 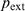 represents the probability that the activation occurred through some external means outside the chain of cause and effect within the cascade, or simply noise.

Equation 2 also allows for the reconstruction of networks in situations when the cascades are recorded in continuous time and when the magnitude of the individual node activities are different, in which case 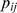 are adjusted using some function 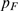 to account for differences in times and amplitudes, i.e.,
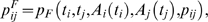
(3)where 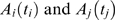 are the amplitudes of events occurring at time 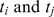 respectively. In the current work we treat the cascades as a pure point process, and ignore the effect of the amplitudes.

Often, neither all of the 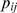 nor the precise function 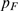 are known and the branching dynamics might be replaced with its mean-field approximation, 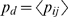. The term “mean-field” used here should not invoke the mean field theory (or self-consistent field theory) in statistical mechanics, but rather its more general meaning, designating any approach in which the actual probability density function 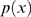 is replaced by the delta function located at its mean value, 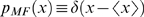. In such case, calculations are much easier and the probability of observing the target node 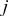 being active at time 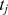 is simply given by
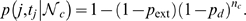
(4)which now depends only on 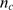.

#### Iterative Bayesian (IB)

Using the IB approach, we attempt to reconstruct a network represented by a set of 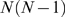 probabilities, 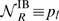, with 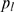 being the probability that a given link 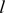 exists. From these individual link priors, the prior probability for a particular network configuration 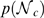 is obtained by
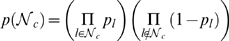
(5)where the product on the left contains 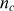 terms and the product on the right 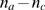 terms. Knowing both the priors and the dynamics terms, the posterior probabilities, 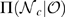, for each configuration 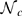 can be calculated using Equation 1. The posterior probability for a particular link 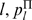, is obtained by summing 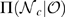 over those configurations 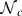 that contain the link 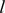 (see Figure 1D and 1E),
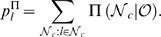
(6)The subset of links in 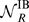 that participated in the current STES will be updated with their posterior values, i.e. 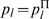, essentially modifying the priors used in the next STES. Initially, the link priors, 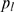 are assigned some small value for all links, and then this iterative procedure is continued until all target nodes are exhausted.

By examining the Eqs. 5 and 6, one can see that the links that acquire a probability 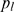 of 0 or 1 will remain at these probabilities. To avoid this, link probabilities 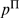 smaller than some prescribed threshold value 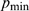 are set to 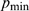. The minimal threshold value is usually chosen to be equal to the initial small prior probability assigned to each possible link and is on the order of 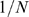. In the presence of noise, the upper boundary 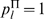 cannot be reached. If the final posterior probability for the existence of a link is higher than some threshold 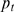, the link is significant, otherwise it doesn't exist. A natural choice for the threshold is 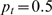.

#### Posterior weighted averaging (with equal link priors)

A shortcoming of the IB is that it weighs heavily recent events, while early data are likely to be ignored. This can lead to reconstruction errors if sudden bursts of noise, in particular towards the end of an experimental observation, are encountered. As an alternative to the IB, we developed the PWA approach. Here, at each STES, we start with the same, pre-assigned, set of prior link probabilities, 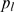. For convenience, we assume that no *a priori* knowledge about the network 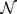 exists and hence make all link priors equal to some fixed value 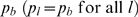. Then, we obtain the posterior probabilities 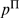 at each individual Bayesian STES for 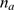 links according to Equation 6. We then derive a weighting factor which is used to combine the individual STES in order to obtain a global measure of connectivity between any two nodes. In order to find the proper weighting factor, we note that when the posterior probability for a link is equal to the prior probability, i.e. 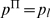, no information is gained and we assign zero weight to such a case. When the posterior probability is 1 we set the desired weight to 1. The suggested weighting factor can then be written as
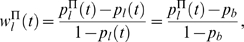
(7)where the index 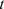 now enumerates different STES, or, in the case of binned data, different time bins.

When 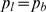, the prior for a given network configuration 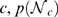, can be written as a function of the number of existing links, 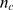, and the number of active source nodes, 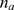,
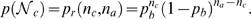
(8)Based on Equation 1, the posterior probability of a particular network configuration 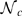, that has 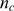 existing links out of 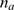 possible links, 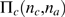, can be written as
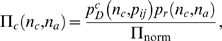
(9)where 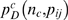 is the dynamics term for the configuration 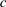 (Equation 2), and 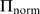 is the normalization term
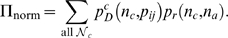
(10)The 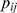 in Equation 9 indicates that 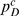 depends on the individual activation probabilities 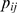 between 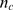 active nodes and the target node 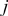.

The posterior link probability for a given link 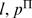, is then the sum of all 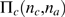 for configurations 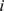 that contain 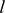. This can be written as
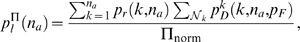
(11)where the second sum goes over all network configurations 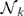 for which 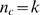 and which contain the link 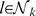, that is, over all possible configurations of the remaining 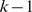 remaining existing links and 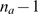 active source nodes. Since links can have different activation probabilities, the second sum in Equation 11 cannot be simply enumerated and full and tedious evaluation of the expression is needed. However, in the simpler case of equal activation probabilities (Equation 4) the second sum in Equation 11 contains 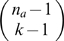 equal terms, i.e.,
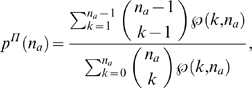
(12)where the dynamic and the prior terms are compounded into 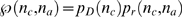. Using Eqs. 4, 8, and 12, a closed form expression for the weights in Equation 7 becomes
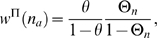
(13)where 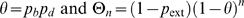. We will use this expression to develop a nonparametric network reconstruction algorithm in the next section.

#### A nonparametric normalized count approach

While the Bayesian approaches allow for the best possible estimate at each step, it requires that the prior probabilities as well as the probabilistic rules of the dynamics are known. Unfortunately, these assumptions are often too strong in real-world situations and we therefore aim to develop a network reconstruction algorithm in conjunction with the Bayesian approach that (a) relies on little or none *a priori* knowledge about the system, that is, it is potentially nonparametric, (b) is efficient, i.e. simple and easy to implement yet robust in the reconstruction, and (c) is not prohibitive for large networks. We will apply this nonparametric method directly to the time binned neuronal activity cascades in which optimal binning width 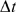 is used [30]–[32], so that the 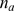 source nodes for a target node within bin 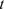 are identified by the active nodes in the preceding time bin, 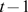 (see the Discussion section for an extension to the continuous time dynamics).

The structure and the dynamics are related as is the case for a critical cascade dynamics on a network. Using this we develop a nonparametric approach for network reconstruction. Assuming that a critical branching process is observed for 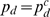, the average node degree, 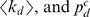 are related by 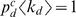. Furthermore, the edge density, or sparsity of the network, 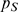 provides a natural choice as the best guess for the uniform link prior, 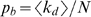, suggesting that 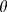 in Equation 13 can be written as 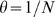. In order to extend the use of this algorithm to subcritical and supercritical regimes, we use the branching parameter, 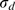, which determines the dynamical regime, with 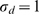 for critical dynamics, and 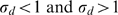 for subcritical and supercritical dynamics respectively. Thus, 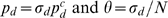. We set 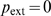 since the actual level of noise is typically not known and to account for noise we will use pairwise shuffling (see Methods). Since the number of nodes, 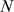, will be known, the weighting factor (Equation 13) now becomes a function of 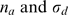 only
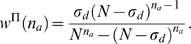
(14)


This expression can be simplified further, if the dynamics is “sparse” so that the cascade activity at any time bin does not consume a large portion of the whole network 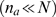. This is a reasonable assumption for a branching process in which 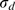 is not much larger than 1. We approximate Equation 14 in two ways. The first one keeps the parameter 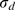,
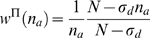
(15)and the second one is nonparametric,
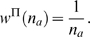
(16)


Generally, 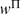 can take on negative values, however, in Equation 15, the negative values are an artefact of the approximation and we set 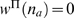 whenever 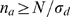. A rough estimate of 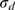 can often be obtained from the observed data, which then renders even Eqs. 14 and 15 nonparametric, but with a caveat that the measured branching parameter might also be influenced by the network topology and thus differ from the purely dynamical 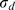. In networks in which the distribution of the node degrees is rather narrow, however, the two values will agree well.

In principle, the PWA method can be applied to any local measure (at the level of a single STES). In the present study, we use the coincidence count between successive time steps, 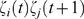, for each link 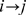, where 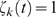, if an event occurred on node 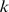 during the time bin 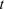, or 0 otherwise. While the coincidence count is a measure of correlation, it does not measure the actual traffic in a network. In order to estimate the topology 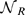, as well as the traffic 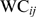, we apply PWA that is we take the average of all one-step estimates weighted by 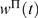 for a given link 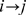. This yields the general expression for the weighted count 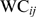,
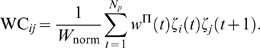
(17)


Here, we are not concerned with the overall scaling factor for 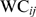, except for making it independent of 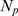, and we use 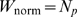. To make 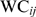 less dependent on particular weighting scheme one can use 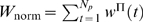.

By replacing the weighting factor 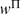 in Equation 17 with those in Eqs. 14 through 16 we obtain three efficient and simple nonparametric measures, which we label 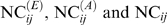 respectively. The expression for 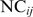 is particularly simple and is the basis of the NC approach:
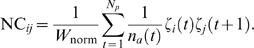
(18)Thus, situations with a large number of potential source nodes are weighted less in the reconstruction process. The only parameter needed is the bin size, for which an optimal value can be found independently as described in [30]–[32], hence it is a nonparametric approach.

#### Other nonparametric approaches

A naive nonparametric approach that one can take to identify directed influence is simply to wait for the instances where exactly one source node is active assuming that active nodes in the near future are causally related to this ancestor. This *single source* (SS) approach, although simple, is useful to establish a reference for computationally more elaborate methods. This approach employs only a subset of all observations, thereby increasing the likelihood of missed links in the network, which reduces the efficiency of the network reconstruction. As will be shown, the SS approach is also prone to large errors in the presence of noise.

Alternatively, the correlation in activity between nodes is commonly used to reconstruct networks from the observed dynamics. It requires a significance threshold, i.e., the expected correlation produced only by chance obtained from either theoretical predictions or by applying nonparametric randomization techniques in order to make a decision whether a particular correlation between two nodes is significant, thus establishing the existence of a link. Here we use the *frequency count* (FC) approach for which all occurrences of successive node activations are counted,
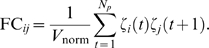
(19)The FC is directly related to correlation, or conditional probability, depending on the normalization factor used. Here we use 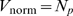. Like all correlation based techniques, the FC approach suffers from the problem of assigning the correct causal structure when multiple source nodes are encountered.

### Shuffling

For each pair of nodes, we can determine some scalar measure of connectivity. For example, these can be node to node correlations, or the FC approach (Equation 19), or the NC approach using 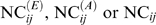 Ultimately, we are trying to use these estimates as a measure of directed influence or causal traffic for each link in the underlying network. However, these measures will also include a contribution from non-causal correlations arising when pairs of nodes are active close in time but had a common ancestor at some prior time during the cascade, or share common inputs directly. We thus have to determine the statistical significance for each of the scalar connectivity estimates. The null-model is obtained by randomizing the recorded activity cascades using *constrained pairwise shuffling*. In this randomization procedure, the times of two randomly selected, active nodes 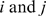 will be switched, such that the node 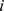 active at time 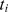, will be assigned time 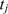 and vice versa.

This shuffling method is straightforward to implement for continuous time events, in which case the time interval distribution will be preserved. For binned data, one will encounter situations where the time bin 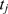 already has node 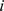 active, and vice versa, in which case the shuffle is aborted and a new pair of nodes is sought. Shuffling in this way preserves the average activity at each node as well as the occupation of time bins with active nodes and thus the dynamical regime of the underlying branching process (see Results). To obtain the resampled dataset, the pairwise switching is repeated 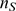 times, 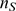 being comparable to the total number of active nodes in the dataset.

By repeating this procedure, 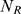 resampled datasets are obtained, each with its corresponding 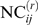 estimate. We use the distribution of the 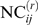 to determine the threshold value 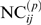 for the given significance level 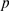. The number of shuffled replicates used to obtain the connectivity estimate at a significance level 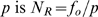, where 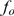 is the “over-shuffling” factor, usually 5 or 10. We obtain the topology, i.e., the adjacency matrix of the estimated network at the significance level 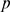 as
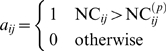
(20)and the architecture as
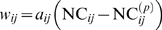
(21)Hence the reconstructed network is a weighted, directed graph, 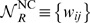, which depends on the prescribed level of confidence, and is supposed to be a measure of causal traffic in the network. Note that by using shuffling, we can determine a separate threshold for each link, thus reducing the bias towards more active nodes and reducing the contribution from correlations in the absence of interactions. When comparing reconstruction results using shuffling and individually derived thresholds with results based on a single common threshold in order to determine the significance of links, we always used the best possible (oracular) single threshold, since in our simulations the original network was known. We also investigated in our simulations if the threshold 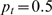 in the IB approach is indeed optimal and it turns out that choosing 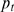 anywhere in the range between 0.1 and 0.9 yields very similar estimates.

### Simulation of Network Topology and Architecture

We simulated the branching process dynamics on 4 different network topologies ranging from a random connectivity with low clustering to a small-world connectivity with high clustering [44]. For the Erdös-Rényi (ER) network, 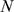 nodes were connected randomly with fixed probability 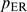 resulting in an average node degree 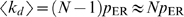 and randomly assigned link directionality. In the Watts-Newman (WN) network [45], each node had 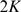 outgoing links to its 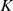 nearest neighbors, after which new links were added randomly with probability 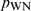 to introduce long-range connections. This algorithm produces a small-world topology with a high clustering coefficient and an average degree 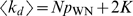 similar to the topology described by Watts and Strogatz [44]. In our simulations we used 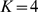. Neither the ER nor the WN topology take into account that many networks self-organize and expand through growth, e.g. cortical neuronal networks. We therefore also tested two growth models that achieve a small-world topology with high clustering coefficients. The Barabasi-Alberts (BA) [46] model uses a preferential attachment rule in which the probability of attachment from a new node is proportional to the node degree of the existing nodes. Each new node establishes 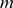 new outgoing links starting initially with 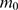 disconnected or fully connected nodes. The resulting topology is scale-free in which the degree distribution decays according to a power law with a slope of −3. Here we use 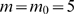 and an all-to-all connectivity for the initial network seed. The BA model requires a new node to attain some knowledge about the degree distribution in the network, which might pose a problem for large networks. In contrast, spatial growth networks [47] do not require global information about the existing network during development. We used the Ozik-Hunt-Ott (OHO) network [48], which is initialized with 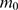 nodes on a circle and all-to-all connectivity. In this network, a new node, whose location is chosen randomly on the circle, attaches preferentially to its 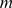 nearest neighbors with outgoing links, hence its growth rule is named geographical preferential attachment. The OHO network is not scale-free, but has a clear small-world property with a high clustering coefficient 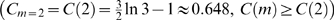 that is independent of the number of nodes. Its average node degree is simply given by 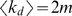 for large networks. In our simulations, we used 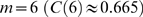. The initial seed for the OHO network is the 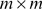 network with an all-to-all connectivity. We note that for both growth models the number of outgoing links was 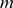 for each node and that both models incorporate a subnetwork (the initial seed) with maximal clustering that is particularly difficult to reconstruct in the supercritical dynamical regime.

For each topology, we created specific network architectures by using constant individual link activation probabilities 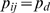, or alternatively, by drawing from a uniform distribution, or truncated Normal distributions (e.g. 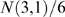 truncated within the range [0,1] and then scaled to 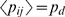).

Different dynamical regimes for each topology were explored on networks with 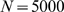 nodes and an average node degree of 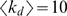. The quality of network reconstruction as a function of reconstruction algorithm, network topology, and network architecture was studied using 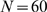 nodes and 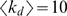, which approximates the number of electrodes from planar integrated micro-electrode array recordings for neuronal avalanches and the corresponding node degree. For the BA and OHO network, the average degree is discretized since it directly depends on the integer parameter 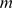, (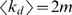 for undirected case). Here we used 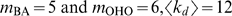.

### Simulation of Network Dynamics

The branching process dynamics was simulated as follows. A source node 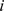 was selected randomly according to some initiation probability distribution (see below) and activated. In the next time step, all outgoing links emanating from 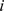 will have a chance to activate its neighbors 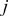 (targets) with the corresponding link activation probability 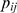. Each activated target now becomes a source for the next generation of active nodes, and this is repeated for successive time steps until no active nodes are found. Heterogeneity in node initiation was simulated by assigning the node initiation probability from a truncated Gaussian profile, 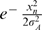, where 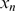 is the normalized set of ordered node indices so that all nodes span the profile from 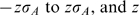 is the heterogeneity parameter. Thus, the probability of choosing the center node (the most active one) was a factor of 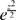 times larger than the probability of choosing the two edge nodes (the least active ones). We used 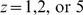, hence the ratios were ≈1.65, 7.4, 2.7×10^5^ respectively.

**Figure 2:**
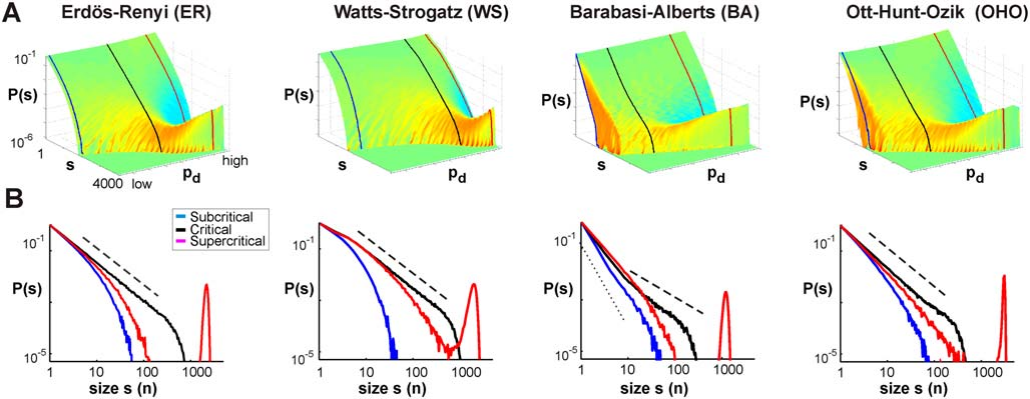
Cascade size distributions obtained from simulations of subcritical, critical, and supercritical branching process dynamics on different network topologies. (A) 3D plots of the avalanche size distributions ranging from the subcritical (low 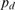, front) to supercritical (high 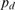, back) dynamics of the branching processes simulated on 4 different network topologies with 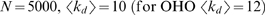. The link probability 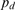 is constant for all links, and served as a control parameter for different dynamical regimes. (B) Size distributions for all 4 topologies at three particular values of 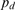, subcritical (blue), critical (black), and supercritical (red) regimes (see the indicated cross-sections in (A)). The distribution of avalanche sizes 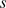 in the critical branching process regime follows a power law with slope of −3/2 (dashed line). A clear critical point is observed for ER and WN networks, while for OHO and BA a power law was observed only for a portion of the size values but not for the full range.

We evaluated three different dynamical regimes of the branching process. In the critical regime, one active node at time 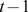 on average will lead to exactly one active node in the next time step 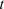 and the distribution of avalanche sizes obeys a power law with a slope of −1.5 [49]. In the ER network, the critical regime is reached if the average link probability 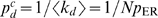, for 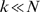 and for WN networks, 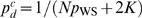. Conversely, sub- and supercritical regimes of the branching process were simulated at 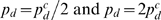, respectively. For the BA and OHO networks, a power law spanning a large range of avalanche sizes was difficult to identify, although their sub- and supercritical regimes were similar to those in ER and WN networks. We therefore used for those simulations a value for 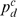 that yielded the closest fit to a power law size distribution between the sub- and supercritical regimes (see also Figure 2). A refractory period ensured that an avalanche ended once, or before, all nodes in the network were activated, a constraint that assured termination of the process particularly when simulating supercritical dynamics.

Random node activation independent from the ongoing dynamics, i.e. due to noise or external inputs, was implemented such that any node on the network could be activated with probability 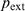 per time step, expressed as 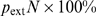. We used a level of 20% for all simulations with noise, which translated on average into the random activation of one node every five time steps, independent from the ongoing dynamics. Note that randomly activated nodes did not initiate new cascades, otherwise they would increase reconstruction efficiency since the patterns of activity in the ‘noise-induced’ cascades would also be influenced in the same manner by the underlying network that we are trying to reconstruct. While noise was used universally, in some instances we also tested the robustness of the algorithms to time jitter, implemented such that every active node at time 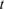 was displaced into time bin 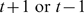 with 20% chance.

### Reconstructing 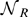 from Cascade Dynamics

We applied the NC, FC, IB, and SS algorithms to different instances of the simulated cascade dynamics on all four network topologies and different architectures. Because the algorithms were described in detail in the Theory section, here, we focus on additional, practical issues.

When reconstructing a network using IB, we used a cut-off value for the number of active nodes considered, 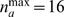, above which the IB iteration is skipped. Those iterations would take a significant portion of the evaluation time and yield only a slight gain in the posterior probability. While this diminished somewhat the performance of the IB particularly in the supercritical regimes, larger values of 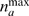 would have resulted in impractically long reconstruction times.

In order to establish significance for various network parameters, we used two randomization techniques, the Erdös-Rényi randomization (ER) and the degree sequence preserving randomization (DSPR) [50],[51]. In ER randomization, links were completely randomized in order to obtain an ER network with an equivalent number of nodes, links, and weight distribution as in the original network. This randomization destroys any correlations and changes the node degree distribution. In the DSPR, two directed links were chosen randomly between four different nodes, and then the target nodes of the two links were switched preserving the degree distribution. This is repeated many times, and in our implementation the number of such switches is equal twice the number of the links in the network (number of links that have not been switched even once is less than 2%).

Finally, for each of the network reconstructions, the total error, 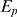, was expressed as the number of links that differed between the reconstructed network 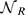 and the original network 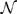 relative to the total number of links in 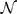,
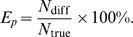
(22)This error counts both false positives, i.e. an estimated link does not exist, as well as false negatives, i.e. an existing link was not identified, and because 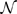 is usually sparse, the error can far exceed 100% of the true number of links. The error was averaged over 10 different realizations for each topology and expressed as mean±standard deviation, if not stated otherwise. When comparing two networks, neither of which represents the “gold standard”, we use the following two measures for comparison. One is, 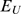, the percent difference in topology, similar to 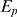, but now expressed as the total number of the differences relative to the number of the links that exist in either of the two networks. This is a less stringent measure than the 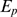, and the maximal error is limited to 100%. The second is the Pearson correlation coefficient between the link weights among the common links in the two networks, 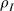, or alternatively, among the links that are in either of the two, 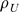.

In order to reduce a potential bias in reconstruction efficiency from arbitrarily selecting a particular significance level, we chose the best reconstruction obtained from the significance levels 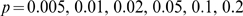. Using an over-shuffling factor of 10, best reconstructions for NC and FC were generally obtained at 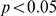. In our simulations, we can also measure the traffic of causal activations through any given link by summing all the activations that actually occurred between its source and target nodes. The resulting traffic for each link was compared with the reconstructed link weights (see Equation 21) to study traffic estimates using FC and NC.

### MEA Recording and Neural Avalanches

Coronal slices from rat dorsolateral cortex (postnatal day 0–2; 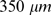 thick) were attached to a poly-D-lysine coated 8×8 multi-electrode-array (MEA; Multichannelsystems, Germany) and grown at 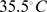 in normal atmosphere in standard culture medium without antibiotics for 4–6 weeks before recording (for details see [29]–[32]). In short, spontaneous avalanche activity was recorded outside the incubator in normal artificial cerebrospinal fluid (aCSF) under stationary conditions (laminar flow of 1–2 ml/min) for up to 10 hrs. For long-term, pharmacological experiments a second set of cultures was recorded inside the incubator (for details on long-term recording conditions see [29]). In short, MEAs with cultures were placed onto storage trays inside the incubator, which were gently rocked (≈200 s cycle time). For recording, single cultures grown on the MEAs for 5–6 weeks were placed into a head stage (MultiChannelSystems, Inc.), which was affixed to a second tray within the incubator and which had the exact same motion as the primary storage tray. This allowed recording from cultures inside the incubator in culture medium under conditions identical to growth conditions. Bath application of the AMPA glutamate-receptor antagonist 6,7-dinitro-quinoxaline-2,3(1H,4H)-dione (DNQX, 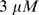 Sigma) was used to reduce synaptic excitability in the cortical network. DNQX was directly added to the culture chamber. For wash, the 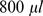 medium was replaced with normal pre-conditioned culture medium. Analysis was based on the following time periods of spontaneous activity: 2–5 hr before, 15–20 hr during DNQX and 2–5 hr after 19 hr of washing of the drug.

Spontaneous local field potentials (LFP) were low-pass filtered at 50 Hz and sampled continuously at 1 kHz at each electrode. Negative deflections in the LFP (nLFP) were detected by crossing a noise threshold of −3 SD followed by negative peak detection within 20 ms and nLFP peak times and nLFP amplitudes were extracted. Neuronal avalanches were defined as spatiotemporal clusters of nLFPs on the MEA. In short, a neuronal avalanche consisted of a consecutive series of time bins with width 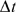 that contained at least one nLFP on any of the electrodes. Each avalanche was preceded and ended by at least one time bin with no activity. Without loss of generality, the present analysis was done with bin width 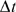, estimated individually [30]. 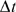 ranged between 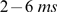 for different sets of cultures. Avalanche size was defined as (1) the number of active electrodes that constitute an avalanche, i.e. the number of nLFPs, and (2) as the sum of absolute nLFP amplitudes on active electrodes. In the former case, size ranged from 1 to 60 (corner electrodes were missing on the array), whereas in the latter case size ranged from 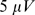 (lowest detection level of an nLFP) up to several thousands of 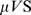.

## Results

### Dynamical Regimes and Cascade Size Distributions

During activity cascades, an active node on average can activate less than 1, exactly 1, or more than 1 node in the next time step in correspondence to the subcritical, critical, and supercritical dynamical regime of a branching process. We therefore identified these three dynamical regimes for each of the 4 topologies by calculating the corresponding cascade size distributions on networks with N = 5000 nodes, 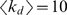 and a constant activation probability 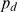 for all links. For both the WN and ER networks, the critical probability, 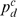, was characterized by a cascade size distribution that followed a power law with a slope of −1.5 as predicted by theory [49] (Figure 2; 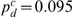 for ER; 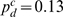 for WN). Conversely, an exponential distribution characterized the subcritical regime in which most cascades engaged only few nodes, whereas in the supercritical regime, a bimodal size distribution revealed that cascades stayed either relatively small or engaged most of the network. For the BA network, the distribution of cascades sizes in the subcritical regime followed a power law with a slope of ≈−3 for sizes <10, suggesting that cascades in that regime were dominated by the degree distribution (slope −3). In contrast, the supercritical regime was identified by a bimodal size distribution. At the transition to the supercritical regime, the BA network revealed a power law slope close to −1.5 for a small range of avalanche sizes (10 to 100 at 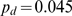), which we used to identify the critical dynamics. For the OHO network, a critical regime was indicated at 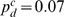 (mean field prediction was 0.085) at which the cascade size distribution revealed a corresponding power law with slope of −1.5 (Figure 2), from which it deviates for large cascade sizes. Thus, given the constraints of a constant 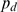, the critical regime in the current simulations represented an approximation of a true critical dynamics for both the BA and OHO network (Figure 2).

The characteristic size distributions for each dynamical regime suggest a varying efficiency in reconstructing networks based on the observed activity cascades. For the subcritical regime, we expect fewer ambiguous situations with multiple source nodes (Figure 1C) and thus better accuracy in network reconstruction. These smaller cascades, however, contain fewer links that can be estimated per unit time, which should slow the reconstruction progress. The opposite holds for the supercritical regime where large cascades allow for a larger percentage of links to be estimated per unit time, while the reconstruction accuracy might decrease due to an increase in ambiguous situations. Consequently, we expect the critical dynamical regime to achieve a balance between these opposing tendencies in network reconstruction. Additionally, in subcritical regime much greater number of initial events will not propagate at all, in which case a reconstruction step cannot be performed. Thus, it takes much longer time to collect the same number of STES in the subcritical regime than it does in critical or supercritical regimes.

### NC Robustly Reconstructs ER Networks for All Dynamical Regimes

**Figure 3:**
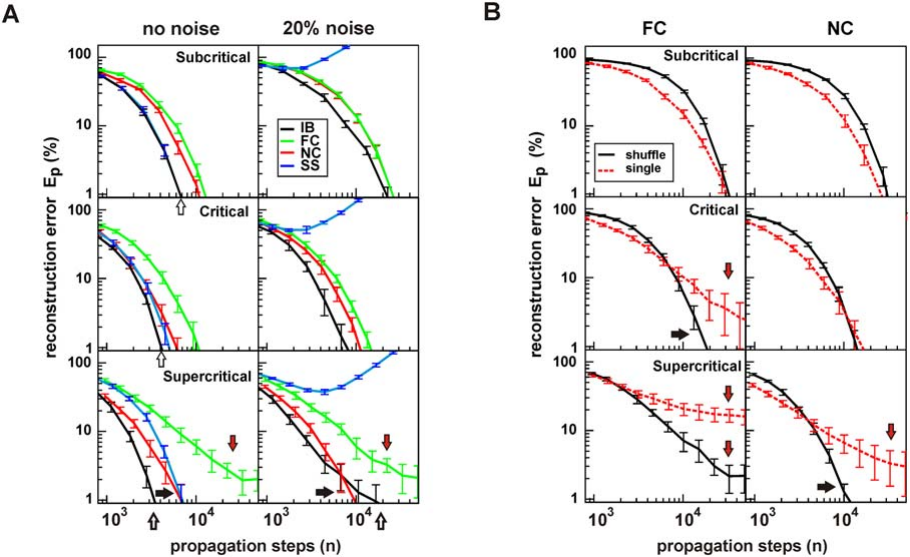
The NC algorithm (NC) performed robust and with high accuracy in both critical and supercritical regimes even in the presence of noise. (A) Reconstruction error 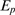 relative to the original ER network for the four algorithms and all three dynamical regimes 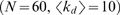. Left: without noise. Right: with 20% noise. Open arrow: total number of reconstruction steps to reach 1% accuracy for the IB algorithm (benchmark). (B) The NC algorithm in combination with shuffling is required to reconstruct ER networks for all three dynamical regimes. 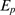 is plotted against the number of propagation steps without shuffling (red broken line, single) and with shuffling (black solid line, shuffle) for the FC (left) and NC algorithm (right). Same network condition as in (A) with 20% noise. Note the robust performance of NC with shuffling in the supercritical regime. Arrows: reconstruction failure (red), success (black), see text. SS: Single Source. FC: Full Count. NC: Normalized Count. IB: Iterative Bayesian. Mean and SD obtained from 10 network simulation replicates.

**Figure 4:**
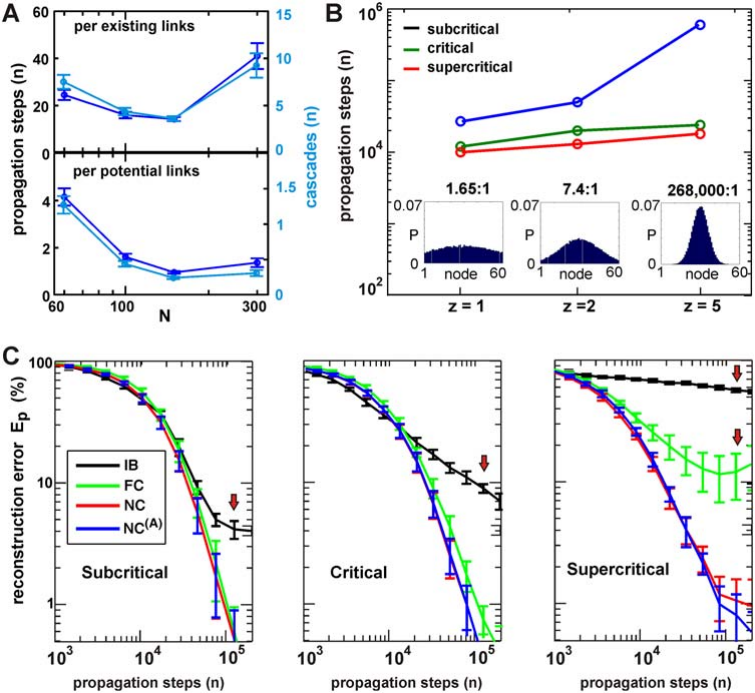
Robustness of the NC for heterogeneous network dynamics. (A) NC scales well with an increasing number of nodes 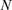 in terms of number of propagation steps, 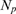 (dark blue) and cascades (light blue) needed to reconstruct network topology within 1% accuracy. The number of needed steps 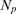 (cascades) is normalized by the number of existing links (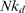, top) and the total number of potential links (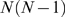, bottom). (B) Network reconstruction in the face of large heterogeneities in node initiation distribution 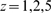. Performance measured in terms of number of propagation steps to reach 1% 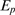 (ER network with 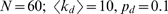, 20% noise). Inset in B: Density plots of node initiation probability for 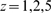). (C) Comparison of IB, FC and NC, when the actual activation probabilities 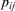 are heterogeneous (normally distributed with 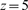) and temporal jitter is introduced in the cascading dynamics (node activation has a 20% chance to be shifted to (t−1) or (t+1)). Note that IB fails to reconstruct networks under these condition. FC is robust but only in the subcritical and critical regime. In contrast, NC and 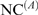 perform robustly for all regimes. Red arrows: reconstruction failure.

We quantified the relationship between the dynamical regime and the reconstruction efficacy by plotting the total reconstruction error 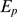 as a function of number of propagation steps, 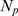, which is the total number of successive time bins that both contain at least one active node. This was done for all three regimes and all four algorithms (Figure 3; ER topology, 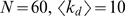, uniform link activation probability 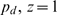 for avalanche initiation; see also Figure 4B). For both FC and NC, the significance of a link was based on 1000 shuffles. For the IB algorithm, the correct value of 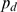 was used in the dynamic term (Equation 4).

In our initial evaluation without noise, the IB algorithm was superior in reconstructing the network in all three dynamical regimes. As predicted from the cascade size distributions, its reconstruction efficiency was higher in the critical regime compared to the subcritical regime (Figure 3A, left, open arrows). Importantly, the IB algorithm further improved in the supercritical regime demonstrating its robust handling of situations with common inputs, where it achieved a high efficiency that is 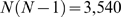 possible links were estimated in approximately the same number of propagation steps in order to reach a reconstruction accuracy of 1%. Similarly, the correlation algorithm FC, while being less efficient than the IB algorithm, faired better in the critical regime when compared to the subcritical regime. However, it failed in the supercritical regime to achieve 1% accuracy even for up to 10^6^ propagation steps demonstrating its sensitivity to correlations due to common inputs (Figure 3A, left, red arrow). Importantly, our newly developed NC algorithm clearly overcame the weakness of the FC algorithm and demonstrated its efficiency in all three regimes (Figure 3A, left, black filled arrow). We note that the error reported is calculated with respect to the number of existing links in the network, i.e. ≈600 links for 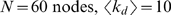 out of 3,600 possible links. Hence a reported error of 1% is equivalent to about 1/6 = 0.167% overall error in deciding whether a link existed or not.

The simple SS algorithm, by avoiding ambiguous situations, performed surprisingly well for all regimes and was comparable to the performances of the IB and NC algorithm. However, the SS algorithm was highly sensitive to noise and relied on the assumption that the observed activations completely arose from the intrinsic dynamics. In fact, when we repeated our simulations in the presence of 20% noise (Figure 3A, right), SS failed entirely in all regimes resulting in errors significantly larger than 100%. Equally important, the IB algorithm now required 4–5 times more propagation steps to reach an accuracy of 1% in the supercritical regime; a sensitivity to noise that originated from the iterative development of the priors over time (Figure 3A, right, open arrow). In the presence of noise, only the NC algorithm robustly reconstructed networks with similar efficiency in the critical and supercritical regime thereby performing even better than the IB in the supercritical regime (Figure 3A, right). In comparison to the standard correlation approach, the NC algorithm provided about 50% improvement in the critical regime and more than a 10-fold improvement to achieve 3% accuracy in the supercritical regime.

These results demonstrate that NC performed best given (1) its simplicity, requiring no assumptions about the network connectivity or network dynamics, (2) its high accuracy for all three regimes, and (3) good reconstruction efficiency of about 2.7 propagation steps per potential link (total 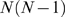 links) for the critical and supercritical regime at 1% reconstruction error.

### Improvement in Network Reconstruction Using Pairwise Shuffling

Correlation methods in network reconstruction commonly utilize a single, global threshold to identify links, e.g. links are assumed to exist for all pairwise node correlations that are above a minimal correlation value (e.g. [18], [20], [52]–[54]). However, heterogeneous node activation frequencies, as well as other conditions, might require different significance thresholds for each link. For the networks in Figure 3, we compared the efficiency in network reconstruction when establishing link significance using either shuffling or, alternatively, a fixed, best possible threshold for both the FC and NC algorithm in the presence of 20% noise. While shuffling performed slightly worse in the subcritical regime, it significantly improved reconstruction accuracy in the critical and supercritical regime (Figure 3B). For the FC algorithm, shuffling was necessary for an accurate estimation in the critical regime, but it was insufficient in the supercritical regime where the error 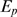 remained high above 1%, even for large numbers of propagation steps (Figure 3B, red arrow). For the NC algorithm, shuffling was required to accurately reconstruct a network with supercritical dynamics (Figure 3B, black arrow). The results, here plotted for 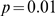, were similar for 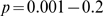 (data not shown). This analysis clearly demonstrates that correlation based methods benefit from using shuffling estimates for thresholds in the critical regime. On the other hand, the NC algorithm in combination with shuffling is required for network reconstructions in the supercritical regime.

The reconstruction results were obtained on a relatively small network with 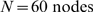, and a question arises on how well it performs for larger networks. Since the network model we are trying to reconstruct has 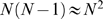 binary parameters, it is natural to expect that the number of needed samples, i.e. propagation steps, for the same reconstruction error should at least increase proportionally to 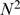. Using NC to reconstruct an ER topology from the cascades in the critical dynamical regime, we demonstrate (Figure 4A) that the number of propagation steps required for 1% reconstruction accuracy scales approximately linearly with the total number of potential links in the network, i.e. it scaled as 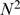, making it a potentially useful algorithm for reconstructing larger networks.

Of particular concern for network reconstruction are situations in which nodes rarely participate in cascade initiations. For example, initiation sites of neuronal avalanches differ up to an order of magnitude in avalanche initiation rate [29],[32]. Such heterogeneity should make it more difficult to reconstruct the topological neighborhood of less active nodes. Nevertheless, as shown in Figure 3B, the NC algorithm accurately reconstructed networks with heterogeneities in node initiation frequency up to a factor of 268,000∶1 for all three dynamical regimes and with only a slight increase in computation for critical and supercritical regimes.

Finally, we tested the robustness of the IB, FC and NC algorithms in reconstructing networks with heterogeneous activation probabilities 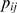 even though the reconstruction algorithms assume a fixed 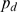 In addition, we introduced a temporal jitter of 20% when binning activity cascades as to account for temporal imprecision in cascade measurements. As before, the noise level was 20% and the node initiation heterogeneity was set to 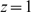. Under these conditions, the IB failed (Figure 4C) to reconstruct the networks to 1% accuracy for all dynamical regimes. Similarly, FC was robust in subcritical and critical regimes, but it failed to reach below a 10% error in the supercritical regime. In contrast, NC always reached below 1% reconstruction accuracy, and performed the best in all regimes. The performance of NC can be further improved in supercritical regimes when the knowledge of the branching parameter, 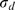 is taken into account, as in 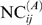 (Figure 4C).

### Efficiency of NC To Reconstruct Different Network Topologies

**Figure 5:**
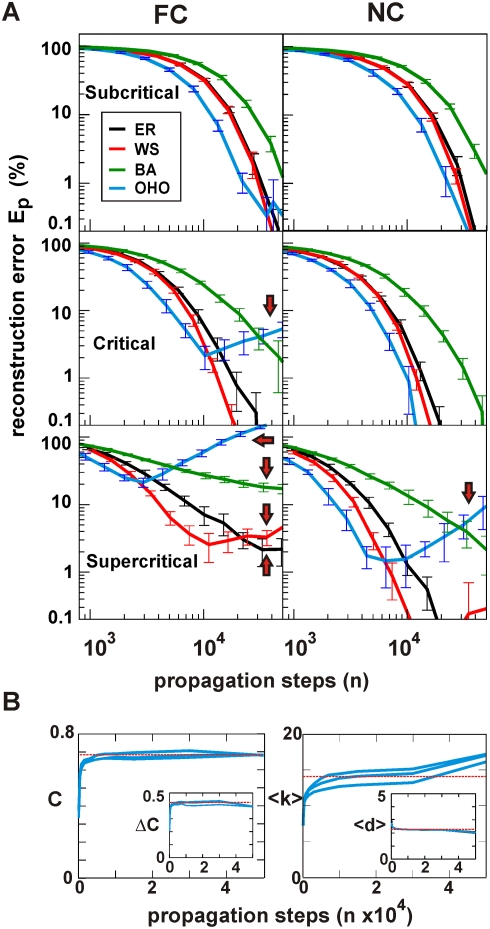
The NC algorithm reconstructs random and most small-world network topologies in all three dynamical regimes. (A) Comparison in reconstruction efficacy between the FC (left) and the NC algorithm (right; 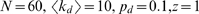, 20% noise). 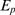 is plotted against the number of propagation steps. FC fails to reconstruct the OHO topology in the critical regime and any of the small-world topologies in the supercritical regime (red arrows). In contrast, NC robustly performs in the critical and supercritical regime for most topologies. Note plot of 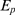 down to 0.1%. (B) Effect of large 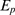 on basic network properties. The clustering coefficient 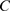 and excess clustering coefficient 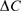 (inset) approach the real values (broken red lines) with increasing number of propagation steps (left). NC reconstruction of the OHO network in A in the supercritical regime. Right: Corresponding analysis for 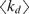 and mean path length 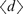. The increase in 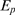 above 30,000 propagation steps barely affects 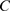, despite slightly increasing 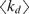 and decreasing 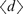. (n = 3 network simulations).

The NC algorithm also allowed for a robust and accurate reconstruction of network topologies that differed from random connectivity. We tested its performance for 4 different topologies and all three dynamical regimes in comparison to the FC algorithm (Figure 5; 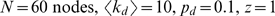 and reconstructed with 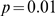 and 1000 shuffles). While the FC algorithm failed for the OHO topology in the critical regime, the NC algorithm reconstructed all topologies in the subcritical as well as critical regime (Figure 5B). Significantly, the FC algorithm failed to reconstruct any of the small-world topologies in the supercritical regime, while the NC algorithm reconstructed the WN as well as the BA network, demonstrated here up to an accuracy of 0.1%. Only the OHO network provided a limit above 1% in the efficacy in network reconstruction (Figure 5B). This limit most likely arises because a supercritical dynamics will engage all nodes most of the time in a highly clustered manner at which pairwise shuffling becomes too constrained (i.e. shuffling two active nodes between two different time points). The errors due to reconstruction will most likely be false positives and random in nature. Hence the overall network parameters (average clustering coefficient, mean path length, average degree) might or might not be affected significantly by the errors of this order of magnitude. Accordingly, we plotted the reconstructed network parameters as a function of propagation steps for the OHO network in the supercritical regime. As can be seen from Figure 5B, even seemingly high error rates of 10% did not significantly affect the clustering coefficient, while the average degrees are biased to larger values, indicating that most of the errors are false positives.

### The Reconstruction of Network Traffic Using NC

**Figure 6:**
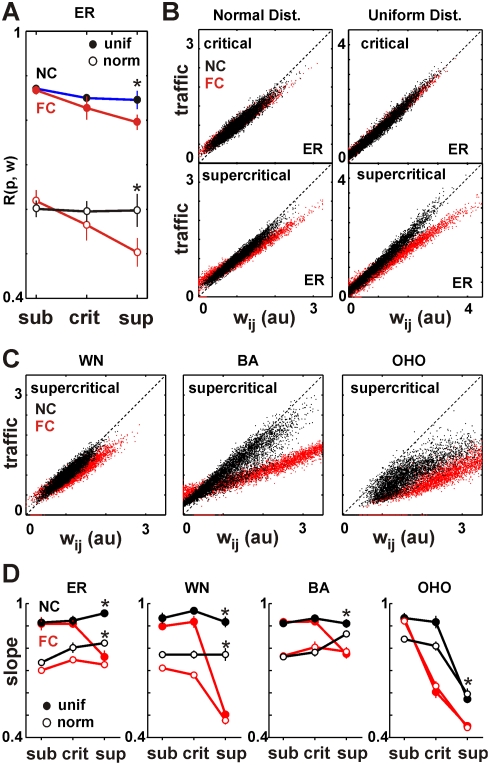
NC improves the reconstruction of the network traffic for different topologies and critical and supercritical dynamical regimes. (A) NC significantly improves the estimation of link activation probability 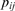 based on link traffic 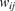. Correlation between 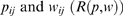 plotted for all three dynamical regimes. The mean and SD for each point were obtained from 10 network realizations. The figure indicates significant improvement by NC over FC for uniform (unif) and normal (norm) distributed 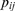 (ER network). (B) NC accurately estimates link traffic 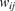. Scatter plot of 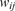 vs. actual traffic for each link for critical and supercritical regimes and two different link activation probability distributions. Note that 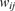 as estimated by NC (black dots) are located along the diagonal (broken line) indicating correct estimates of local traffic (n = 10 networks combined). (C) Same as in (B), plotted for WN, BA, and OHO topologies in the supercritical regime with normally distributed 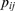. (D) NC estimates the network traffic more accurately than FC. Slope of linear regression taken from analysis as shown in (B) and (C) for all topologies, two distributions, and three dynamical regimes. Note that the slope for NC is closer to 1, compared to FC in particular for the critical and supercritical regime. Reconstruction accuracy is low for OHO in the supercritical regime (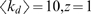, 20% noise). Results taken after 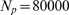.

The traffic on a network, i.e. the network flow, is one of the most important aspects that characterizes network functionality [55]. It was reliably estimated by NC for all three dynamical regimes and most topologies. We studied the correlation between the known link activation probabilities 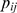 and the estimated link weights 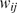 on an ER network for which link activation probabilities were drawn either from a uniform distribution or a truncated normal distribution between [0,1] with 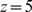 (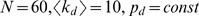, and 20% noise). In Figure 6A it is shown that for both uniform and normal distributed activation probabilities, NC did significantly better than FC in relating the reconstructed weights 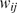 to the original weights prescribed as 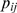, particularly in the supercritical dynamics. Furthermore, when correlating the estimated 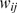 with the actual traffic in the network, calculated during the simulation, we found that NC provided a very good measure of the traffic between two nodes (slope close to 1; Figure 6B and 6C). In contrast, FC significantly underestimated the traffic for increasingly higher traffic values (slope ≪1). These results, obtained on an ER network topology, were also confirmed for small-world topologies, where NC reliably estimated the traffic on the WN and BA network for all three dynamical regimes. Only for the supercritical regime on the OHO network did the NC algorithm estimate the traffic poorly (Figure 6D, black dots). However using 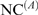 further improved the reconstruction in traffic similar to that of an equivalent ER network (R = 0.72; data not shown).

### The Small-World Topology of Neuronal Avalanches

**Figure 7:**
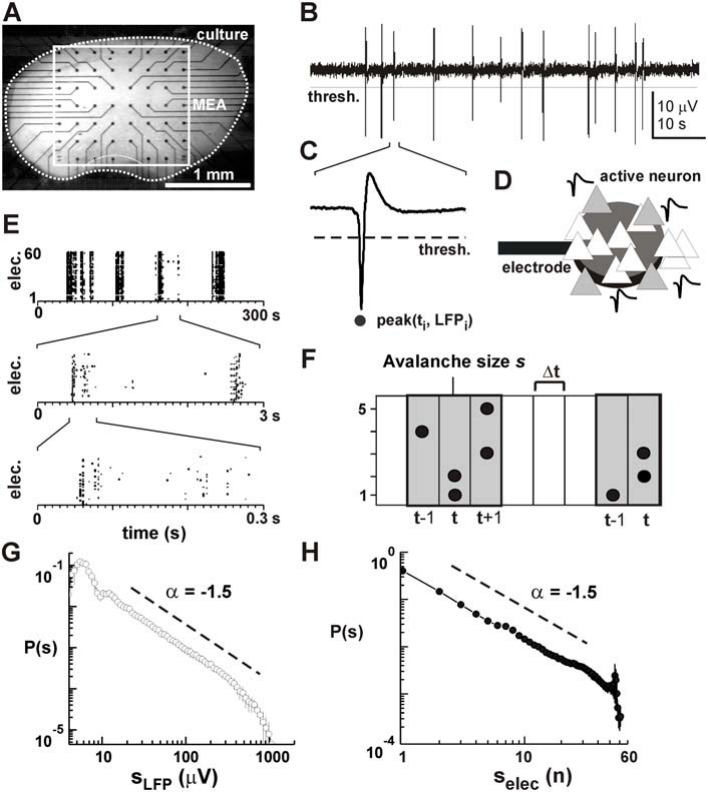
Neuronal avalanches in organotypic cortex cultures recorded with integrated planar micro-electrode arrays (MEA). (A) Light microscopic image of an organotypic culture from rat somatosensory cortex grown for 12 days on an MEA (square). Electrode positions are visible as an 8×8 dot matrix with connecting leads attached. (B) Spontaneous activity at a single electrode is characterized by the occurrence of large deflections in the local field potential (LFP). (C) A single LFP deflection at higher temporal resolution taken from (B). The negative peak deflection (nLFP) that crosses a negative threshold (broken line) is characterized by its peak amplitude 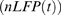 and peak time 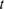. (D) The nLFP can be interpreted as the local synchronized activity of a subgroup of active neurons (gray triangles) recorded by a nearby electrode (disc). (E) nLFPs on the MEA are clustered into periods of high activity separated by periods of relative quiescence (top), an organization that repeats at higher temporal resolutions (middle and bottom). (F). Sketch of the definition of neuronal avalanches using 5 electrodes on the MEA. A neuronal avalanche arises from the concatenation of successive time bins of width 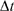 that contain at least one nLFP. (G,H) Power law in avalanche size distribution with slope of −1.5 for sizes expressed in summed absolute nLFP amplitudes (left) or number of active electrodes, i.e. nLFPs (right; 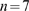 networks; recalculated from [30]).

**Figure 8:**
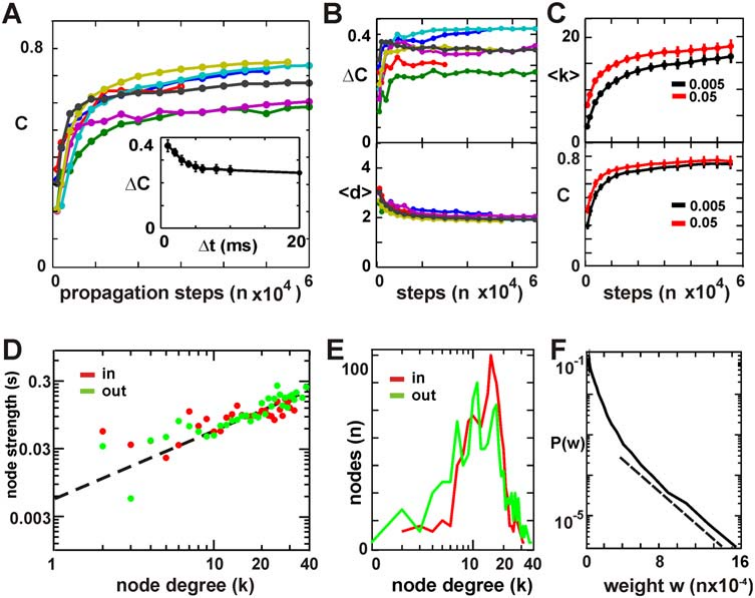
Neuronal avalanches reveal a functional small-world topology. (A) The clustering coefficient 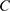 converges to a high and constant value with increasing number of propagation steps for all 7 networks studied. Inset: Average excess clustering coefficient 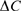 for all networks and different 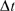. Note that 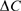 does not represent an error of reconstruction, but an important network property that measures specificity in network clustering. The quality of reconstruction should be judged by whether or how fast it approaches the correct steady value (B) Corresponding change in 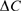 (top) and mean path length 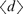 (bottom)) for the networks reconstructed in A. Small-world topology is defined by a high excess clustering 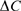 and low 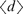. (C) Average change in mean node degree 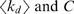 for two significance values in link reconstruction. Note robustness of 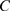 to an increase in significance whether link exists. (D) The node degree is linearly related to the node weight for both in and out degrees. Broken line: slope = 1. Each network reconstructed at 30,000 steps with links smaller than 20% of the maximal traffic pruned. (E) Node degree distributions for in and out degrees reveal a predominance of nodes with 10–20 links. (F) Semi-logarithmic plot of link weight probability demonstrates the presence of an exponential decay (broken line) for links with high traffic. (D–F) All networks from (A) combined.

Given that the avalanche dynamics can be realized on different topologies (see Figure 2), we used the robust performance of the NC algorithm for different dynamical regimes and widely varying network topologies in order to reconstruct the functional topology and architecture of real neuronal networks that display neuronal avalanches recorded with integrated planar micro-electrode arrays (MEA) from neuronal cortex cultures. Spontaneous activity in these cultures is characterized by negative deflections in the local field potential (nLFP) indicative of a local synchronization within a subgroup of neurons near the electrode (Figure 7A–D; [30]). The organization of nLFPs in the neuronal network takes on the form of complex spatiotemporal patterns that evolve over successive time bins (Figure 7E and 7F). These patterns, when interpreted as successive node activations (see Figure 1B), were used to reconstruct the functional network topology and network architecture. Under normal conditions, the dynamics that emerges in this system [29] is characterized by neuronal avalanches whose sizes obey a power law with a slope of −1.5 for avalanche sizes measured in terms of integrated nLFP amplitude or number of nLFPs indicative of a critical state (Figure 7G, [6],[56],[57]). Importantly, the power law in avalanche sizes correlates with a sequential activation of local neuronal groups that is analog to a critical branching process [29]–[32]. In the absence of any knowledge of the real underlying network organization, we reasoned that the reconstructed network architecture might be reliable if its features converged with increasing number of propagation steps in the reconstruction process, e.g. as shown for the simulated OHO network in Figure 5B. Indeed, the network parameters such as the clustering coefficient, 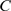, and average node degree, 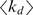, remained largely constant beyond 30,000 propagation steps. This was in agreement with our simulation results, where NC achieved a smaller than 1% error estimate for all topologies in the critical regime within a similar range of propagation steps (Figure 5). Importantly, despite the relatively small network size of 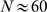 and an average degree of 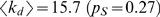, the clustering coefficient of 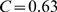 was significantly higher than what would be expected for corresponding randomized versions of the network 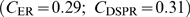. Similarly, we also plot the excess clustering 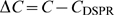, a network parameter (not a reconstruction error in 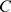) that measures the clustering coefficient in the network that is beyond the one of an equivalent randomized version of the network. Results for 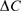 indicate that the high clustering coefficient was not simply due to saturation by adding more and more links into a small network (Figure 8A and 8B). These networks have nearly a linear relationship between the node degree and its strength, i.e. the summed weights of all links at a node, 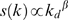, with 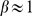 (Figure 8D) while Figure 8E shows the node in- and out-degree distributions 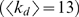. The weight distribution of the links revealed an exponentially decaying tail demonstrating the presence of a few links with large traffic (Figure 8F).

Given that the relatively high clustering was achieved with a small network diameter of 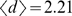 (Figure 8A and 8B), which was similar to those of the equivalent randomized networks 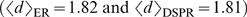, our findings demonstrate that the neuronal cultures with neuronal avalanche dynamics establish a small-world topology as previously reported in abstract form [58],[59]. The functional network topology of the cortex *in vitro* cultures (and acute slices [31]) derived from neuronal avalanches is compared to the results reported for various neural systems in Table 1. The networks range from full brain and cortical networks among different anatomical and functional areas of the brain [16], [44], [60]–[63] to cortical slices and cultures, as well as the neural network of the nematode C-elegans [44]. The table also shows the results for 21 cortical networks binned at 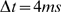 (14 were acquired in the course of the previous studies, and combined with the current set of 7, also re-binned to the same 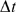). The networks and the sources of this data are listed in the caption. One should note that these networks, with exception of the C-elegans are not very sparse, in which case the clustering coefficient will depend on the size of the network, as the table roughly indicates. A better comparison between these different systems can be achieved by using the excess clustering 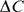, found in the range between 0.13 and 0.32, and which shows no obvious dependence on network size or sparsity.

**Table 1:** Network properties for some known neural systems: C-elegans [44]; Rat Cortex *in vitro*, Acute Slice (average of 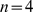 datasets, binned at 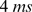) [31]; Macaque Brain [61]; Macaque Visual and Somatosensory Cortex [61]; Cat Brain [62]; Rat Cortex *in vitro*, Cultures 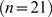, binned at 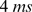 [29],[30]; Rat Cortex *in vitro* Cultures 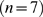, gathered at 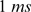 and re-binned at 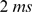 [30]; Cat Cortex [62]; Macaque Visual Cortex [60].

Network	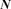	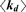	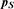	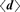	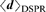	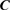	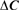
C-elegans	297	7.9	0.03	4.00	3.06	0.24	0.15
Rat Acute Slice (n = 4)	50	5.6	0.12	2.94	2.40	0.33	0.19
Macaque Brain	71	10.5	0.15	2.34	2.06	0.51	0.26
Macaque (Vis-SM)	47	10.7	0.23	2.05	1.90	0.61	0.23
Cat Brain	95	22.4	0.24	1.92	1.81	0.54	0.15
Rat Culture (n = 21)	58	15.7	0.27	2.14	1.81	0.55	0.25
Rat Culture (n = 7)	59	15.8	0.27	2.21	1.81	0.63	0.32
Cat Cortex	52	15.7	0.31	1.81	1.71	0.60	0.19
Macaque Visual	32	9.8	0.32	1.76	1.71	0.59	0.13

The networks are ordered by increasing sparsity 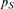.

### Correspondence between Functional and Structural Small-World Topology

Functional connectivities are dynamically modulated even on a millisecond time scale [21],[22]. For example, the functional connection of a single synapse, i.e. its efficacy to elicit a spike in a post-synaptic neuron, depends on the depolarization of the post-synaptic neuron, which itself is linked to the neuron's inputs from within the network, i.e. level of network activity. This suggests that the functional small-world topology reconstructed from the dynamical cascades, which captures the spatiotemporal organization of spiking activity [33], might change with a change in network activity. On the other hand, local synaptic plasticity mechanisms such as spike-timing dependent plasticity [64] are expected to translate successive neuronal activations as reflected in the spontaneous dynamical cascades into a corresponding increase in synaptic strength thereby establishing a structural correlate of the observed dynamics. In that case, the network organization might be expected to be relatively robust to a decrease in overall activity levels.

**Figure 9:**
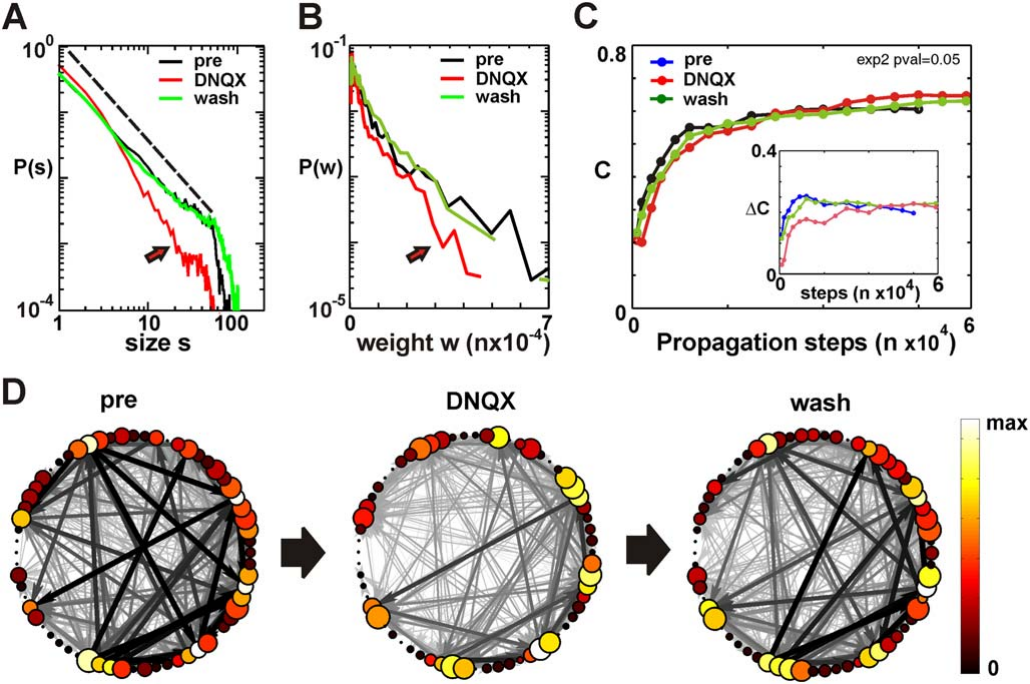
Functional small-world topology derived from neural avalanches is robust to an acute reduction in network traffic. (A) DNQX, which reduces excitatory synaptic transmission between neurons, changes the avalanche size distribution from a critical (power law, broken line −1.5) to a subcritical (exponential) dynamics in which the presence of large avalanches is significantly reduced (red arrow, single network). Distributions are calculated before (pre), during reduced excitation (DNQX), and 24 hr after recovery (wash). (B) DNQX also reduces the traffic in the network (arrow). (C) The clustering coefficient derived from a critical neuronal avalanche dynamics is similar to that derived in the presence of 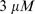 DNQX, (single network). 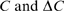 (inset) is plotted against the number of propagation steps. (D) Directed, weighted architecture for the network in (A) reconstructed at 35,000 propagation steps for all three conditions. Node degrees (color, node diameter) and link weights (low: gray; high: black) were scaled between maximum and minimum values of the pre condition respectively. Note the similarities in the existing links and the node degrees for all three conditions despite significantly lower link weights in the presence of DNQX.

By taking advantage of the NC algorithm to reconstruct network architectures in subcritical and critical regimes, we tested the robustness of the functional small-world topology to acute changes in network activity. We acutely reduced the efficacy of excitatory glutamatergic fast synaptic transmission in the cultured networks by bath application of the AMPA receptor antagonist DNQX (n = 3 networks). As expected, 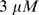 of DNQX significantly reduced the rate of spontaneous cascades by 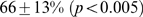. Thus, in order to compensate for the reduced number of propagation steps per time, networks were reconstructed from ≈20 hr of activity in the presence of DNQX compared to 2–5 hrs of the control and wash condition. DNQX also reduced the formation of large avalanches leading to size distributions more similar to that of a subcritical state, which clearly deviated from the power law with a slope of −1.5 for the pre and wash condition (Figure 9A). DNQX significantly reduced the traffic on the network, which under normal conditions revealed an exponential distribution (Figures 8 and 9B). Despite these significant reductions in cascade rate and size as well as link traffic, the small-world topology of the critical network obtained before and after DNQX, nevertheless, was reliably reconstructed during DNQX as indicated by the similarity in the clustering coefficient 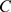 with increasing number of propagation steps (Figure 9C). On average, 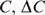, as well as 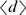 was not different between controls and DNQX 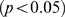. A detailed link-by-link comparison using 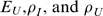, between the “pre”↔“wash” showed an error of 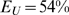 and correlations, 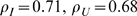. Similarly, a comparison between “pre”↔“DNQX”, and “DNQX”↔“wash” yielded 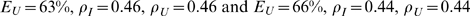, respectively. When the comparison were made between the randomized versions of each network (ER randomization), the results were virtually the same for all three cases, 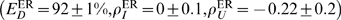. These results show that while these networks are far from identical, their overlap is significantly larger than expected by chance.

## Discussion

In the present study, we developed a method that derives a weighted directed graph based on the observed cascade dynamics, which successfully overcomes ambiguous source and target node correlations in all dynamical regimes of a branching point process. Several methods have been previously employed to cope with the issue of common inputs when using a correlative approach. For example, using delayed correlations, Cecci et al. [20] demonstrated power law scaling in human fMRI data even when links with zero delays indicative of common input were removed. A three-node motif approach using mutual information allowed to remove potential links arising from common input resulting in undirected small-world graphs reconstructed from spontaneous spiking activity in dissociated cultures [52]. Assuming an Ising-model underlying pairwise node correlations, non-directed functional connections have been estimated for networks of up to 10 nodes from spontaneous neuronal activity *in vitro* [65],[66] and genetic interactions [67]. Although, the last approach is able to identify common input situations, it results in non-directed graphs, in contrast to our approach which also reconstructs directed network traffic.

### Bayesian Approaches to Network Reconstruction

The Bayesian approaches described here differ from the so-called Bayesian networks, or belief networks [68]–[70], which specialize in the reconstruction of directed, acyclic graphs with a smaller number of configurations to be explored. In order to reconstruct cyclic graphs, “loopy” Bayesian network approaches [71] can be used, however, they are, even in their approximate form, NP-hard [72]. Bayesian networks are particularly useful in small networks when precise Bayesian inference is required for each link. In contrast, the IB or PWA approaches in the present study are meant for the reconstruction of large networks from large datasets. For that purpose we derived and tested new methods for reconstructing the functional network topology and traffic from dynamical network cascades. We made the Bayesian methodology feasible by dividing the observations and the network into individual target activations with the corresponding active subnetworks (STES). The essential computational reduction was achieved by using the assumptions of (a) only the events in the near past (the source nodes) are a potential cause for an activation event in the cascade and (b) the activation events of two different target nodes that have common source nodes are independent. Both assumptions make sense in neuronal networks such as the cortex, in which events in the near past predominantly influence the present state of a neuron and where the synaptic transmission of a neuron at different postsynaptic sites is independent. All these methods rely on the assumption that the underlying dynamics is stochastic. A fully deterministic dynamics would not allow to discriminate direct from indirect influences.

To combine individual STES and to obtain the reconstructed network, 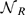, we used the IB and PWA approach. They enable one to improve the reconstruction reliability whenever additional knowledge about the dynamics (or priors in the case of PWA) becomes available. They are computationally feasible, since their computational complexity is simply the number of STES, 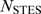, times the complexity of the individual STES. We will assume that the 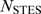 needed in an observation for a given reconstruction accuracy is 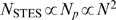 (as was found for NC, see Figure 4A). Hence, the complexity of the IB is 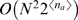, where 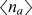 is the average number of 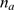 over all STES. It will be likely that 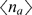 is a function of 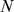 in the critical and supercritical regimes, but less so in the subcritical regime. When 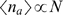, the exponential complexity 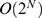 of IB can be managed to some degree by introducing a cut-off value, 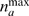, thus reducing the complexity to 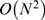, but keeping a large pre-factor 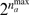. The computational complexity of individual STES in PWA will in most cases be equal or less than 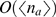. For NC, the individual STES have complexity 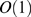, hence, the NC has the same low complexity as FC and other correlation methods, 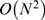, but it produces much better estimates of causal traffic and connectivity, making it a candidate algorithm for the reconstruction of large networks. Note, that most of the computational demand in NC comes from shuffling, whose complexity also is 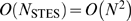. Technical considerations of this algorithm are discussed in the next paragraph (see also Text S1 for the implementation summary).

The PWA approach can also be extended to include situations when the cascade propagation speed is highly heterogeneous, i.e. the continuous time approach is necessary, and/or when the amplitudes of the events need to be considered. This will require some knowledge, or experimental estimate, on how temporal differences and event amplitudes will affect the activation probabilities (see Equation 3). In these cases, the equivalent of the expression in Equation 16 becomes
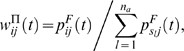
(23)where 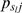 is the link activation probability for the link connecting the 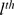 active source node 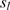 and the target node 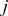. This expression is obtained in the limit of 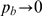. A simple inclusion of the weights can also be obtained by treating 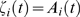, in which case 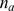 is not the number of active nodes but the total strength of the sources 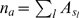. This more general framework, requiring the simulation of continuous time dynamics and varying amplitudes was beyond the scope of this manuscript.

Although PWA was derived from Bayesian considerations, strictly speaking it is not a Bayesian method, particularly not the NC algorithm. When PWA uses uniform priors, one can argue that it is essentially a maximum likelihood method. The difference, however, with the maximum likelihood approach is that we use uniform priors on the links, but not the configurations themselves, which are the elements of our sample space. Thus, different configurations will get assigned different prior probabilities. When the prior probabilities for the existence of any link 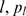, are small, or are assigned based on the sparsity 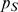 of a network, the existence of a link can be established using a nonparametric measure similar to correlation. Historically, arguments have been made that, in situations where prior knowledge is not available, a precise choice of the prior probability is not crucial [73] as long as the choice is smooth in the region of high likelihood. Thus, a uniform and sufficiently small probability will lead to essentially the same final estimate [74].

### Technical Considerations of the NC Algorithm

The general methodology of PWA and IB was derived in our Theory section. We then tested a particular nonparametric instance of PWA, the NC algorithm, with the goal of reconstructing large networks from large records of a point process dynamics. The NC is essentially a weighted correlation measure, with the weight inversely proportional to the number of potential source nodes. This weighting is not arbitrary, and if one uses a different weighting factor, e.g. 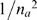, it does not perform as well as NC (data not shown). If one assumes small prior probabilities for each link, this result becomes intuitive, since the posterior probability for the existence of simultaneous links is negligible, hence each link's probability is inversely proportional to the number of possibilities, i.e. active source nodes 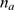. Importantly, we did not assume that 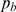 is small, but only that it is equal to the sparsity of the network and that the dynamics is near the critical point. This indicates that the validity of the NC algorithm does not rely on the precise choice of 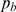. The more elaborate IB approach with fully known dynamics established a benchmark that was closely met by the NC algorithm. The NC algorithm returns the link weights that are an approximate measure of the causal traffic across each link. In this paper we tested, using the simulations of a branching point process on a network, the case when the activation probabilities do not depend on the magnitude of the events and the event times are discrete. More general cases can be addressed using an appropriate activation function in equation 3, and using a different weighting factor for PWA (see Equation 23).

#### The advantages and limitations of NC

*Advantages:* (i) The NC algorithm is nonparametric and requires no prior knowledge of the dynamics, but performs close to the IB approach when the latter fully utilizes that knowledge; (ii) It is computationally as simple as FC and other correlation methods, but produces much better estimates of causal traffic and connectivity, particularly for small-world networks; (iii) the NC algorithm is robust, not only to changes in the dynamical regime, but also to deviations from the dynamical assumptions. The NC algorithm performed well when applied to the branching point process dynamics with large heterogeneities in initiation rate, heterogeneities in activation probabilities, 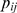, and uncertainty to temporal binning, in contrast to IB and FC (Figure 4C).

*Limitations:* (i) the NC is not as specific, or reliable, as the Bayesian Networks when the existence of a particular link in a network is to be established; (ii) The selection of 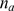 prior events as the potential sources requires some knowledge about the dynamics. The fixed time cut-off, of time-binned events that we use in this work, might fail when more complex temporal dependences between the nodes are encountered; (iii) The NC relies on shuffling to obtain the null-model nonparametrically. However, shuffling can be constrained in certain dynamical conditions, for example, in the subcritical and supercritical dynamical regimes (see the subsection on Shuffling below).

In general, we envision this algorithm to be used for a general network topology when the dynamics of the network is moderately sparse, i.e. when the number of active nodes at any time is not very large, as opposed to correlation based methods which work only when the dynamics is extremely sparse. Note that even a dense network can be reconstructed, provided the observed dynamics is sparse. We expect NC to work well in situations where the correlation methods are commonly used, but with an added advantage that it will be less sensitive to changes in the dynamical regime. The algorithm summary is given in Text S1.

#### The influence of network size and the length of observation on network reconstruction

We note that when increasing the network size while keeping the average degree constant, a network reconstruction error of 1%, which is relative to the existing links in the network, becomes more and more stringent as 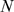 increases and the sparsity 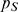 of the network drops 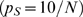. For example, while the error rate per potential link is 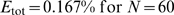, this changes to 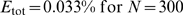. Furthermore, as 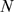 grows the minimal achievable error rate, 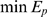, becomes finite and grows as 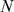 increases. For 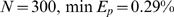, while for 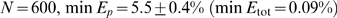. For very large and very sparse networks a different shuffling scheme with additional constraints might improve this accuracy limit. For example, one could consider to partially shuffle the record of dynamical cascades, e.g. where the number of pairwise shuffles is one quarter of the total number of active sites in the dataset i.e. using eight times less pairwise shuffles than the default that we use throughout the paper. In this case, the minimal achievable error for 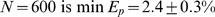, but requires a 20% longer data record (data not shown).

As our results show, the number of needed propagation steps 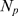 is on the order of few multiples of 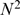. Since the shuffling is not guaranteed to provide an accurate null model, having too many observations will tend to introduce false positives. Thus, of particular concern is the stability of the reconstructed architecture as a function of observation length. Often, reconstructions are done based on the whole, a priori defined length of recording. Robustness can be demonstrated by repeat analysis of subdivisions of the record [75] or devising records of different lengths [10], including the calculation of a cut-off parameter [75]. In the present study, robustness of the reconstructed network was demonstrated by the convergence of a set of network parameters towards a reasonable constant value with increasing number of propagation steps. Naturally, this convergence was particularly robust for the clustering coefficient 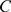, which in contrast to the average degree, is less affected by the erroneous addition of random links. Importantly, this convergence occurred for the neuronal networks at around the same number of propagation steps as was expected from our network simulations and was robust to changes in the dynamical regime. In general, any quantity that is not sensitive to the addition of random links will be robust to the existence of the false positives in the reconstruction.

### Shuffling To Increase Reconstruction Reliability

Shuffling of the original time series is commonly used to establish a priori statistical distributions for the null-hypothesis. Our results clearly demonstrate that pairwise shuffling significantly improves the reconstruction accuracy in the critical and supercritical regime. On the other hand, this method imposes strong limitations resulting in a conservative model that not only maintains the average activity rate of each node, which prevents the introduction of correlations due to rate modulation [22], but also the exact lifetime and size distribution of cascades, thus ensuring that the shuffled raster remains in the same dynamical regime. This shuffling method reaches its limits in the supercritical regime with highly synchronized cascades, e.g. when almost all nodes become active within 1 time step for most cascades, in which the constraints of the pairwise shuffling limit its statistical power. Similarly, pairwise shuffling becomes constrained in the subcritical regime because of the limited number of nodes participating in cascades. Alternative methods combined with pairwise shuffling, such as temporal jittering, using a smaller portion of the raster to determine thresholds, or limiting total number of shuffles, might improve reconstruction efforts further in these cases.

The *ad hoc* use of a global threshold in order to extract a functional connectivity from correlation matrices is often justified by providing a range of thresholds for which the obtained results are robust [18], [20], [52]–[54]. In the present study, we obtained thresholds for each potential link, which significantly outperformed the global threshold approach in the critical and supercritical regimes. The calculation of a probability value using a conservative model, i.e. maintained firing rate and cascade sizes and durations also naturally allows these thresholds to be interpreted in terms of significance for individual link existence. As shown in Figure 8C, topological features were shown to be robust for different significance thresholds.

### Branching Process Dynamics

Our simulation of the branching process incorporated a refractory period during which a node remained inactive before being able to participate in a cascade again. Thus, the simulated dynamics represents a branching process only in the limit of large number of nodes 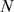. Notably, refractory periods for nodes are common in many real systems, where they arise from energy limitations such as transport capacities and where they serve several major purposes, such as limiting the rate with which each node engages in the network dynamics and terminating cascades in the supercritical regime. In the temporal domain, refractory periods support the formation of non-recurrent dynamics in an otherwise recurrent network. For example, in neuronal networks, each neuron after its action potential is not responsive to the near future neuronal feedback [76], or in epidemics [9] typically studied in Susceptible-Infected-Removed models [77], in which infected individuals acquire immunity against re-infection supporting the view of epidemic spread as an essential forward cascade with little recurrence. While we have addressed the existence of different dynamical regimes on different topologies, we have not studied comprehensively all possible issues that might affect the dynamics of the network, e.g. network modularity [78]. Despite the dynamic feed-forward aspects of most cascades, the resulting functional architecture is not limited to acyclic graphs because potentially recurrent links between nodes that do not engage in one cascade can be active during other times.

### Small-World Functional Topology of Cortical Microcircuits

In the present study, we derived the directed, weighted functional architecture of superficial cortical layers [29],[31] grown on planar integrated micro-electrode arrays. We demonstrated that a small-world functional topology of neuronal avalanches is robust to an acute reduction in network traffic, suggesting that it potentially arises from a corresponding structural small-world topology of cortical micro-circuits.

The neuronal avalanche dynamics that arises in these layers *in vitro* parallels layer formation in the intact animal [33]. The reconstruction of the architecture was based on neuronal avalanches, dynamical cascades that form in analogy to a critical branching process [29],[30] for which our simulations show robust and accurate network reconstruction using the NC algorithm. The estimated clustering coefficient stabilized as predicted from our network simulations. Importantly, a similar topology was recovered from acute, subcritical network dynamics in the presence of DNQX. This suggests that the subgraph described by a cascade does not depend on the overall state of the network, but might underlie structural components of the network as formed by the number and strengths of neuronal connections. A small-world topology combines short distances between network sites with high clustering that allows for diverse functionality of subgraphs, as shown recently for sensory activities in the visual cortex of the cat [79].

Previous studies in dissociated neuronal cultures have quantified dynamical cascades during spontaneous neuronal activity using a variety of measures such as conditional probability [80], pairwise delayed-correlation indices [81], and sequential ordering [82]. Additionally, functional topologies were derived using correlation methods with global correlation thresholds [83]–[85]. As shown in the present study, the correlation approach might not adequately address functional connectivity, particular for dissociated cultures which have been shown to display supercritical dynamical cascades [82]. Despite these potential limitations, correlation and mutual information based methods derived non-directed functional small-world topologies from spontaneous activity in dissociated cortical cultures [52],[86], in line with our topological findings for the neuronal avalanche dynamics in layered cultures. Our study further quantified the network traffic, which was characterized by an exponential tail distribution similar to what has been found for the weight distribution in dissociated neuronal cultures [52] and airport traffic networks [55]. These characteristics of the small-world architecture formed by neuronal avalanches provide important constraints for future simulations of this type of cortical dynamics.

## Supporting Information

Text S1 Supporting Information
(0.06 MB PDF)
