# Supplementary material for: Efficient Network Reconstruction from Dynamical Cascades Identifies Small-World Topology of Neuronal Avalanches
Source: PLoS Comput Biol. 2009 Jan 30;5(1):e1000271. doi: 10.1371/journal.pcbi.1000271 (PMC2615076; doi:10.1371/journal.pcbi.1000271)
Supplement: Text S1 — Supporting Information (0.06 MB PDF) [file pcbi.1000271.s001.pdf]

Supporting Information : Text S1

NC Algorithm Summary

Input: (i) A raster of activation data, recorded as (time,node) pairs, i.e. (t_e, s_e); (ii) p-value for the reconstruction, p.
Output: The reconstructed weighted directed network

1. Determine the rule by which the n_a source nodes will be chosen for every target node. In our current implementation in reconstructing networks from neuronal avalanches, we took the approach developed in [29, 30] in which the events were binned with a time window Δt chosen as a mean of the time-interval distribution from successively activated nodes in the network. In this particular case, only nodes in the preceding time bin are treated as the potential source nodes. Thus, the raster becomes a $N_t \times N$ sparse matrix, where $N_t = T_{exp}/\Delta t$ is the total number of time bins.

2. Determine the number of propagation steps, N_p, i.e. number of the incidences in which each of the two successive time-bins contains at least one active node. This number should be greater than the number of potential links in the network ($N(N-1)$) (Figure 4A)

3. Evaluate the NC_{ij} factor for all links, according to Equation 18. If highly supercritical dynamics is suspected, the more general Eqs. 14 and 15 should be used.

4. Use pair-wise shuffling (subsection "Shuffling" in the Methods) to produce $N_R = f_o/p$ replicates of the raster, where $f_o > 1$ (see Methods). For each replicate r and each link $i \to j$ determine the factor $\mathrm{NC}_{ij}^{(r)}$ using Equation 18. From the PDF of $\mathrm{NC}_{ij}^{(r)}$ and the given confidence level p determine $\mathrm{NC}_{ij}^{(p)}$.

5. From NC_{ij} and $\mathrm{NC}_{ij}^{(p)}$, obtain the reconstructed network topology and weights according to Eqs. 20 and 21.

Additional steps might include validating the reconstruction by varying the length of the raster records used, to ensure that the reconstruction is not dependent on N_p. The implementation of this algorithm was done in C and in Matlab. The pre-compiled version of this code will be made available upon publication at http://mscl.cit.nih.gov/spaj (link to PWAnetrec).

List of Abbreviations

aCSF	—	artificial cerebrospinal fluid
BA	—	Barabasi-Alberts network [46]
DSPR	—	degree sequence preserving randomization
ER	—	Erdös-Rényi network (or randomization scheme)
FC	—	Frequency Count reconstruction approach
IB	—	Iterative Bayesian approach
MEA	—	Multi-Electrode Array (Multichannelsystems, Germany)
NC	—	Normalized Count approach
OHO	—	Ozik-Hunt-Ott (OHO) network [48]
PWA	—	Posterior Weighted Averaging
SS	—	Single Source approach
STES	—	Single Target Estimation Step; a simple Bayesian estimation step consisting of a single target node at a particular time instance and a subset of potential source nodes.
WN	—	Watts-Newman (modified Watts-Strogatz) network

Mathematical Notation

$\mathcal{O}$	—	generic symbol designating an observation, i.e. a pattern of activations
$\mathcal{N}$	—	generic symbol designating a network
$\mathcal{N}_c$	—	instance of network topology (adjacency matrix). For STES it is a particular configuration by which source nodes connect to the target node (Figure 1E)
n_a	—	number of active source nodes considered in STES
$\langle n_a \rangle$	—	average n_a over all STES in a given experiment
n_c	—	number of active source nodes that connect to the target node in a particular $\mathcal{N}_c$ being considered
p_l	—	probability that a given link l exists in IB; also a link prior
p_t	—	threshold for p_l in IB; link exists if $p_l \geq p_t$
p_b	—	uniform prior probability for link existence used in PWA
$p(\mathcal{N}_c)$	—	prior probability of a given network configuration $\mathcal{N}_c$
$p(\mathcal{O}\|\mathcal{N}_c)$	—	likelihood term (here called *dynamics* term) in Bayesian reconstruction
p_D^c	—	dynamics term for a particular configuration $\mathcal{N}_c$
Π	—	generic designation for posterior probabilites
p^Π	—	generic term for posterior probability for links
p_l^Π	—	posterior value of p_l obtained for link l within IB iteration using Equation 6
$\Pi_c(n_c, n_a)$	—	the posterior probability of a particular network configuration $\mathcal{N}_c$, having n_c existing links out of n_a possible links
$\mathcal{P}(n_c, n_a)$	—	compounded the dynamics and the prior terms, $p_D(n_c)p_r(n_c, n_a)$
$\mathcal{N}_R$	—	network obtained through reconstruction from the dynamics

$\mathcal{N}_R^{\mathrm{NC}}$ — network obtained by the normalized-count (NC) approach

$\mathcal{N}_R^{\mathrm{IB}}$ — network obtained by the Iterative Bayesian (IB) approach

$n_a^{\max}$ — cut-off value for n_a in IB reconstruction above which the corresponding STES is skipped

N_p — number of propagation steps in a time-binned raster, i.e. number of times that both of the successive time-bins contain at least one active site

N_{STES} — total number of STES used in reconstruction

t — index into individual time bins, or STES

t_i — time of an event "i" occurred, or for a particular STES, an event that occurred on the ith node

A_i — amplitude of an event "i", or the one occurring on the ith node

p_{ij} — activation probability for the link $i \to j$; a fixed probability that an active source node i will activate its target node j in the avalanche dynamics

$p_F(...)$ — function that represents a-priori knowledge of the continuous time branching process dynamics, and describes how the timing of the events (in most cases only the timing differences, $t_j - t_i$) and amplitudes of the events A affect the original activation probabilities, p_{ij}.

p_{ij}^F — predicted probability for the $i \to j$ activation, obtained using a-priori knowledge contained in $p_F(...)$

p_d — mean-field approximation to our branching process dynamics, $p_d = \langle p_{ij} \rangle$, or just a uniform activation probability

p_d^c — critical value of the probability p_d

w_{ij} — weight of a directed link $i \to j$

a_{ij} — binary indicator of the existence of a link $i \to j$

m ($m_{\mathrm{BA}}, m_{\mathrm{OHO}}$) — number of edges added in growing networks, OHO and BA

m_0 — initial number of nodes in growing networks

K — number of the nearest neighbors ($2K$) that a node connects to in WN network

w^{Π} — weighting factor in PWA

σ_d — the branching parameter, which determines the dynamical regime

Π_{norm} — the normalization term for the posterior probability

$\{s_l\}$ — source node index that connects to the target through the lth link

W_{norm} — normalization factor for PWA weighted measures

V_{norm} — normalization factor for a correlation measure

Δt — the width of a time window at which events were binned

z,σ_A — parameters determining the heterogeneity of the branching process initiation

N — number of nodes in a network

k_d — degree of a particular node

C — clustering coefficient of a network

$\langle k_d \rangle$ — average node degree for the whole network

$\langle d \rangle$ — average node-to-node distance

C_{ER} — C of the randomized network using ER randomization

C_{DSPR} — C of the randomized network using DSPR randomization

p_S	—	edge density, or sparsity of network, defined as $p_S = \#\text{links}/(N(N-1))$
p_{ER}	—	probability that a link exist in Erdös-Rényi network
p_{WN}	—	probability that a random link exist in Watts-Newman network
p_{ext}	—	in our simulations, the probability that the node activation is caused by noise
FC_{ij}	—	frequency of successive activations between the nodes i and j
WC_{ij}	—	weighted measure of successive activations between the nodes i and j
NC_{ij}	—	scalar measure assigned to each link according to Equation 18
$\text{NC}_{ij}^{(E)}$	—	scalar measure assigned to each link, similar to NC_{ij} but with a weighting factor according to Equation 14
$\text{NC}_{ij}^{(A)}$	—	the simplest approximation of $\text{NC}_{ij}^{(E)}$ which keeps the branching parameter σ_d (Equation 15)
$\text{NC}^{(E)}$	—	NC method which uses $\text{NC}_{ij}^{(E)}$ instead of NC_{ij}
$\text{NC}^{(A)}$	—	NC method which uses $\text{NC}_{ij}^{(A)}$ instead of NC_{ij}
p	—	p-value, confidence level in network reconstruction
$\text{NC}_{ij}^{(r)}$	—	NC_{ij} values obtained from surrogate data sets using shuffling
$\text{NC}_{ij}^{(p)}$	—	the threshold value for NC_{ij} at the significance level p, obtained from the empirical distribution of $\text{NC}_{ij}^{(r)}$
n_S	—	number of cascade events switches in pair-wise shuffling
N_R	—	number of replicates obtained with pair-wise shuffling
f_o	—	over-shuffling factor that increases N_R for a given p, for better estimation of $\text{NC}_{ij}^{(p)}$
E_p	—	measure of the reconstruction error, defined in Equation
E_{tot}	—	reconstruction error relative to all possible links, $N(N-1)$
E_U	—	the percent difference between two topologies, quantified as the total number of differences divided by the number of links that exist in either of the two (range: 0-100%)
ρ_I, ρ_U	—	correlation between two architectures using the links that exist in both networks (intersect, ρ_I), or in either network (union, ρ_U)
$E_D^{\text{ER}}, \rho_I^{\text{ER}}, \rho_U^{\text{ER}}$	—	measures E_U, ρ_I, and ρ_U obtained by comparing the ER randomized networks
